# High-Origin Visceral and Renal Arteries with a Symptomatic Right Renal Artery Aneurysm: A Case Report and Literature Review

**DOI:** 10.3390/jcm15197408

**Published:** 2026-09-24

**Authors:** Abdulaziz Althunayan, Othman Alabdullah, Ghadah Alarify, Joud Aldar, Mohammed Almasabi, Kaisor Iqbal, Sultan AlSheikh, Abdulmajeed Altoijry

**Affiliations:** 1 Division of Urology, Department of Surgery, College of Medicine, King Saud University, Riyadh 12372, Saudi Arabia; 2 College of Medicine, King Saud University, Riyadh 12372, Saudi Arabia; 3 College of Medicine, Imam Abdulrahman Bin Faisal University, Dammam 34212, Saudi Arabia; 4 Division of Vascular Surgery, Department of Surgery, College of Medicine, King Saud University, Riyadh 12372, Saudi Arabia

**Keywords:** renal artery aneurysm, high-origin renovisceral arteries, endovascular treatment, aortorenal bypass, open reconstruction

## Abstract

**Background:** Renal artery aneurysms (RAAs) are uncommon visceral arterial lesions whose management depends on symptoms, patient-specific rupture risk, and arterial anatomy. Markedly high origin renovisceral arteries may further complicate endovascular access and reconstruction. This report describes the management of a symptomatic RAA in a young woman with multifocal aneurysmal disease and unusual renovisceral arterial origins at the 11th and 12th thoracic vertebral levels (T11–T12). **Case presentation:** A 35-year-old woman presented with intermittent right loin pain. Computed tomography angiography demonstrated multiple right renal artery aneurysms, the largest measuring 1.7 cm, together with a 0.6-cm left renal artery aneurysm and a 0.67-cm superior mesenteric artery aneurysmal dilatation. The celiac trunk, superior mesenteric artery, and both renal arteries originated unusually high at the T11–T12 level. An initial trial of right renal artery stenting through right brachial access was technically difficult. Selective angiography after difficult cannulation demonstrated contrast extravasation from a minute right renal artery perforation. The patient remained hemodynamically stable; heparin was reversed with protamine, and repeat angiography 5 min later demonstrated complete sealing without further extravasation. The procedure was therefore aborted with close inpatient observation. Definitive treatment was achieved by ligation of the right renal artery aneurysmal segment and a retrocaval aorta-to-right renal artery bypass using a reversed great saphenous vein graft. At six months, the graft remained patent with preserved renal perfusion and no recurrence of the presenting symptom. **Conclusions:** This case demonstrates how extreme renovisceral anatomical variation, multifocal aneurysmal disease, and adverse arterial characteristics can limit the feasibility of endovascular treatment. In selected patients in whom secure endovascular access or renal branch preservation cannot be achieved, open reconstruction remains an effective renal-preserving option. Long-term surveillance is important because of the remaining untreated aneurysmal lesions and the limited duration of follow-up in this report.

## 1. Background

Renal artery aneurysm (RAA) is an uncommon visceral arterial lesion that is increasingly detected with cross-sectional imaging. Contemporary management has moved away from aneurysm diameter as the sole determinant of treatment and instead incorporates symptoms, morphology, location, growth, associated arterial disease, and patient-specific rupture risk [1,2,3,4]. Recent guideline and systematic-review data support individualized selection among surveillance, endovascular treatment, and open reconstruction [1,2,3,4].

Management is particularly important in women of childbearing potential because pregnancy-related hemodynamic and hormonal changes may increase arterial wall stress and RAA rupture during pregnancy can have severe maternal and fetal consequences. The Society for Vascular Surgery guidelines specifically recommend treatment of noncomplicated RAAs in women of childbearing potential regardless of aneurysm size when operative risk is acceptable [1], while more recent vascular guidance also emphasizes pregnancy-related risk and individualized management [3,4].

Treatment feasibility also depends on renovisceral arterial anatomy. Renal arteries usually arise from the abdominal aorta near the first and second lumbar vertebral levels (L1–L2), while substantial variation exists in the level and configuration of renal and major visceral arterial origins [5,6]. A renal artery arising at the lower thoracic level is distinctly uncommon and has mainly been described in case reports [7]. When high arterial origin is combined with sharp angulation, ostial disease, stenosis, tortuosity, or multiple aneurysmal segments, catheter stability and device delivery may become particularly difficult [8,9,10].

We report a 35-year-old woman with symptomatic multifocal right RAA in the setting of an unusual cluster of high-origin renovisceral branches at T11–T12, with additional aneurysmal changes in the left renal and superior mesenteric arteries. The case is notable for the coexistence of multifocal aneurysmal disease and markedly high renal, celiac, and superior mesenteric arterial origins, failure of an initial endovascular attempt, and successful renal-preserving open reconstruction.

## 2. Case Presentation

A 35-year-old female with an unremarkable past medical history presented with intermittent right loin pain as her sole presenting complaint. Upon admission, the patient was afebrile and hemodynamically stable. Physical examination was unremarkable. The abdominal assessment revealed a soft, lax, and non-tender abdomen with no palpable pulsatile masses or organomegaly. Peripheral vascular examination demonstrated intact and palpable bilateral pedal pulses, with no clinical evidence of lower extremity ischemia. Diagnostic workup for this symptom identified a focal aneurysmal dilatation of the right renal artery, prompting referral to vascular surgery.

Laboratory investigations were largely within normal limits. Notably, renal function was preserved, with a serum creatinine of 61 µmol/L and normal electrolyte levels. A complete blood count was unremarkable. Prothrombin time (PT), partial thromboplastin time (PTT), and international normalized ratio (INR) were within normal ranges. Given the complex vascular anatomy, an autoimmune and vasculitis workup was conducted to rule out connective tissue disorders; the results were negative for antineutrophil cytoplasmic antibodies (ANCA) and cryoglobulins, with a non-specific borderline antinuclear antibody (ANA) titer of 1:80.

Computed tomography angiography (CTA) confirmed complex visceral arterial anatomy and associated pathology. The abdominal aorta demonstrated focal wall irregularity and calcification at the T11 level. The celiac trunk, measuring 5.13 cm in length, originated at the T11 level, while the superior mesenteric artery originated at the T11–T12 level. The superior mesenteric artery showed a small aneurysmal dilatation at its origin measuring 0.67 cm, with focal moderate distal stenosis. The left renal artery originated at the upper T11 level and exhibited a tortuous course with proximal wall irregularity and an aneurysm measuring 0.6 cm. The right renal artery originated at the lower T11–T12 level and demonstrated significant pathology, including ostial calcification, moderate stenosis shortly after its origin, and distal tortuosity with multiple aneurysmal dilatations, with the two largest measuring 1.2 cm and 1.7 cm. Representative CTA and volume-rendered images of these findings are shown in Figure 1 and Figure 2.

An initial endovascular trial of right renal artery stenting was undertaken via right brachial access. The right brachial artery was punctured under ultrasound and fluoroscopic guidance, a 5-F sheath was inserted and exchanged for a 7-F 90-cm Destination sheath, and heparin was administered according to protocol. Control angiography demonstrated the anomalous origins of the superior mesenteric and both renal arteries. Cannulation of the right renal artery origin was technically difficult and was achieved using a soft Terumo wire and Burn catheter. Selective angiography then demonstrated contrast extravasation from a minute right renal artery perforation; the patient remained hemodynamically stable throughout the procedure. Heparin was reversed with protamine 20 mg intravenously, and repeat angiography 5 min later showed no further extravasation and complete sealing of the perforation. Because the perforation had sealed and the patient remained stable, the procedure was aborted and close ward observation was undertaken. The operative angiographic assessment described an extremely tortuous right renal artery with a mid-segment aneurysmal dilatation of approximately 1.5 cm; this angiographic estimate is reported separately from the maximal 1.7-cm diameter measured on preprocedural CTA. Post-procedure CT demonstrated a stable right renal artery following the extravasation. Subsequent surveillance CTA also demonstrated a localized aortic dissection with a false lumen and no active bleeding. No separate intervention for the localized dissection was documented in the available operative record. Representative angiographic images of the right renal artery aneurysms are shown in Figure 3.

Ultimately, the patient underwent definitive open surgical repair. A midline exploratory laparotomy was performed from the xiphoid to just above the symphysis pubis, the falciform ligament was excised, and the liver was fully mobilized. The retrocaval right renal artery was identified according to the preoperative CTA, and multiple aneurysmal outpouchings were dissected while the left renal vein was identified and protected. Proximal right renal artery control and distal control beyond the aneurysmal segments were obtained. The left great saphenous vein was harvested from the thigh and reversed. Clamps were applied to the proximal and distal right renal artery and the aorta; the clamp and renal ischemia times were not recorded. A retrocaval side-to-side aorta-to-right renal artery bypass was then constructed using the reversed great saphenous vein graft with 6-0 Prolene. The aneurysmal segment was proximally ligated with large clips, and intraoperative Doppler confirmed graft patency. The procedure was otherwise uneventful. Histopathological examination of a right renal artery aneurysm specimen revealed myxoid degeneration and mixed inflammatory infiltrates consistent with degenerative changes; there was no evidence of malignancy.

The patient had an uneventful postoperative recovery and was discharged on postoperative day 7 on antiplatelet therapy. At the six-month follow-up, the right loin pain had not recurred. Follow-up computed tomography angiography confirmed patency of the aorta-to-right renal artery vein graft with preserved renal perfusion. The untreated left renal artery and superior mesenteric artery aneurysmal changes remained indications for continued vascular surveillance.

## 3. Discussion

This case combines several features that materially influenced management: a young woman of childbearing potential with symptomatic disease, multifocal aneurysmal involvement, and markedly high renovisceral arterial origins. The right renal artery, left renal artery, celiac trunk, and superior mesenteric artery were clustered at the T11–T12 level, creating a vascular geometry substantially different from routine renal artery intervention. The coexistence of multifocal aneurysms in both renal arteries and the superior mesenteric artery further supports careful assessment of the entire renovisceral circulation rather than treating the right-sided lesion as an isolated abnormality.

The indication for treatment was based on clinical context rather than aneurysm diameter alone. Contemporary recommendations emphasize individualized decision-making, particularly for symptomatic patients and women of childbearing potential [1,3,4]. Although many small asymptomatic RAAs demonstrate slow growth and may be managed with surveillance [11,12], the combination of right loin pain and childbearing potential distinguished this patient from a routine low-risk surveillance population.

The anatomy had direct procedural consequences. A high renal artery origin alone does not necessarily preclude endovascular treatment; however, in this patient it coexisted with sharp angulation, ostial calcification, proximal stenosis, extreme tortuosity, and multiple aneurysmal segments. The planned endovascular strategy was a trial of right renal artery stenting via right brachial access. Control angiography confirmed the anomalous superior mesenteric and bilateral renal arterial origins, and selective cannulation of the right renal artery was technically difficult. During selective angiography after cannulation, a minute renal artery perforation with contrast extravasation occurred before the intended endovascular treatment could be completed. The patient remained stable, and repeat angiography after protamine reversal demonstrated complete sealing without further extravasation. This procedural sequence illustrates how unfavorable access geometry and severe tortuosity can compromise catheter control even before definitive device deployment. Contemporary endovascular standards and systematic reviews emphasize that treatment strategy should be matched to aneurysm morphology, branch involvement, landing zones, and the ability to maintain stable access while preserving renal perfusion [8,9].

Endovascular therapy remains an important option for anatomically suitable RAAs and can include coil embolization, stent-assisted techniques, covered stent exclusion, and other reconstructive approaches [8,9]. A recent systematic review and meta-analysis found broadly comparable major outcomes between endovascular and open repair, with shorter hospital stay after endovascular treatment [2]. More recent multicentre data similarly support anatomy-tailored selection, with both open and endovascular approaches achieving high success when applied to appropriate lesions [10]. The present case therefore should not be interpreted as evidence against endovascular therapy; rather, it illustrates its technical limitations when access geometry and arterial characteristics are unfavorable.

Open reconstruction remains an important renal-preserving option when endovascular exclusion is not technically feasible or would compromise important renal branches. In this patient, a midline laparotomy with liver mobilization allowed identification of the retrocaval right renal artery. After protection of the left renal vein and establishment of proximal and distal renal arterial control, a reversed left great saphenous vein graft was used to construct a retrocaval side-to-side aorta-to-right renal artery bypass. The aneurysmal segment was proximally ligated, and intraoperative Doppler confirmed graft patency. This reconstruction provided revascularization beyond the diseased and tortuous segment while avoiding dependence on an endovascular landing zone within the abnormal artery. The operative record did not document the aortic or renal arterial clamp duration. Contemporary comparative evidence continues to support open reconstruction for selected anatomically complex lesions [2,10].

Histopathological examination demonstrated myxoid degeneration with mixed inflammatory infiltrates consistent with degenerative change. Intimomedial mucoid or myxoid degeneration has been described as a rare arterial disorder characterized by mucoid accumulation, elastic tissue disruption, and aneurysm formation [13]. However, a causal relationship between this finding and the extravasation or localized dissection observed during catheter manipulation cannot be established from a single case. The histopathology should therefore be regarded as a possible marker of arterial wall degeneration rather than proof of the mechanism of the procedural complication.

The etiology of the multifocal arterial abnormalities remains uncertain. The negative ANCA and cryoglobulin testing and nonspecific borderline ANA result do not support overt systemic vasculitis, but a single case cannot exclude other non-inflammatory arteriopathies. The coexistence of bilateral renal artery aneurysmal disease and superior mesenteric artery involvement warrants continued clinical and imaging follow-up and should prompt consideration of a broader arteriopathy when clinically appropriate.

The six-month result is encouraging, with a patent bypass and preserved renal perfusion, but follow-up remains short for a young patient with multifocal aneurysmal disease. Longitudinal surveillance is important both for graft durability and for the untreated left renal and superior mesenteric arterial lesions. Current guidelines emphasize continued imaging follow-up after treatment and during conservative management of visceral and renal artery aneurysms [3,4].

This report has several limitations. It describes a single patient and therefore cannot establish comparative effectiveness between open and endovascular repair, define the underlying cause of the multifocal arteriopathy, or demonstrate a causal relationship between myxoid degeneration and procedural arterial injury. Follow-up is currently limited to six months. In addition, the operative record did not document the renal or aortic clamp duration or the specific rationale for choosing right brachial access. The available records also did not provide a specific treatment rationale or formal long-term imaging interval for the untreated left renal and superior mesenteric arterial aneurysmal lesions. The localized aortic dissection was documented on follow-up imaging without active bleeding, but no separate procedural intervention for it was recorded. Longer-term surveillance will be necessary to assess bypass durability, renal perfusion, the behavior of the untreated aneurysmal lesions, and the evolution of the localized dissection.

Overall, the principal clinical lesson is the importance of matching treatment strategy to arterial anatomy and procedural feasibility. Endovascular treatment remains valuable when stable access, appropriate landing zones, and renal branch preservation can be achieved. When these conditions are absent in the setting of extreme renovisceral anatomical variation, open reconstruction remains an essential alternative for definitive aneurysm exclusion and renal preservation.

## 4. Conclusions

This case demonstrates a rare combination of high-origin renovisceral arteries, multifocal aneurysmal disease, and technically unfavorable right renal artery anatomy. The experience emphasizes that treatment of RAA should be individualized according to symptoms, patient-specific rupture risk, branch anatomy, and procedural feasibility. When endovascular treatment is limited by unstable access, adverse geometry, or concerns regarding renal branch preservation, open surgical reconstruction with autologous vein bypass can provide effective renal-preserving treatment in selected patients. The principal limitations of this report are its single-case design and six-month follow-up. Continued longitudinal imaging is required to assess graft durability and the untreated left renal and superior mesenteric arterial lesions, and longer follow-up will help define the durability of this management strategy.

## Figures and Tables

**Figure 1:**
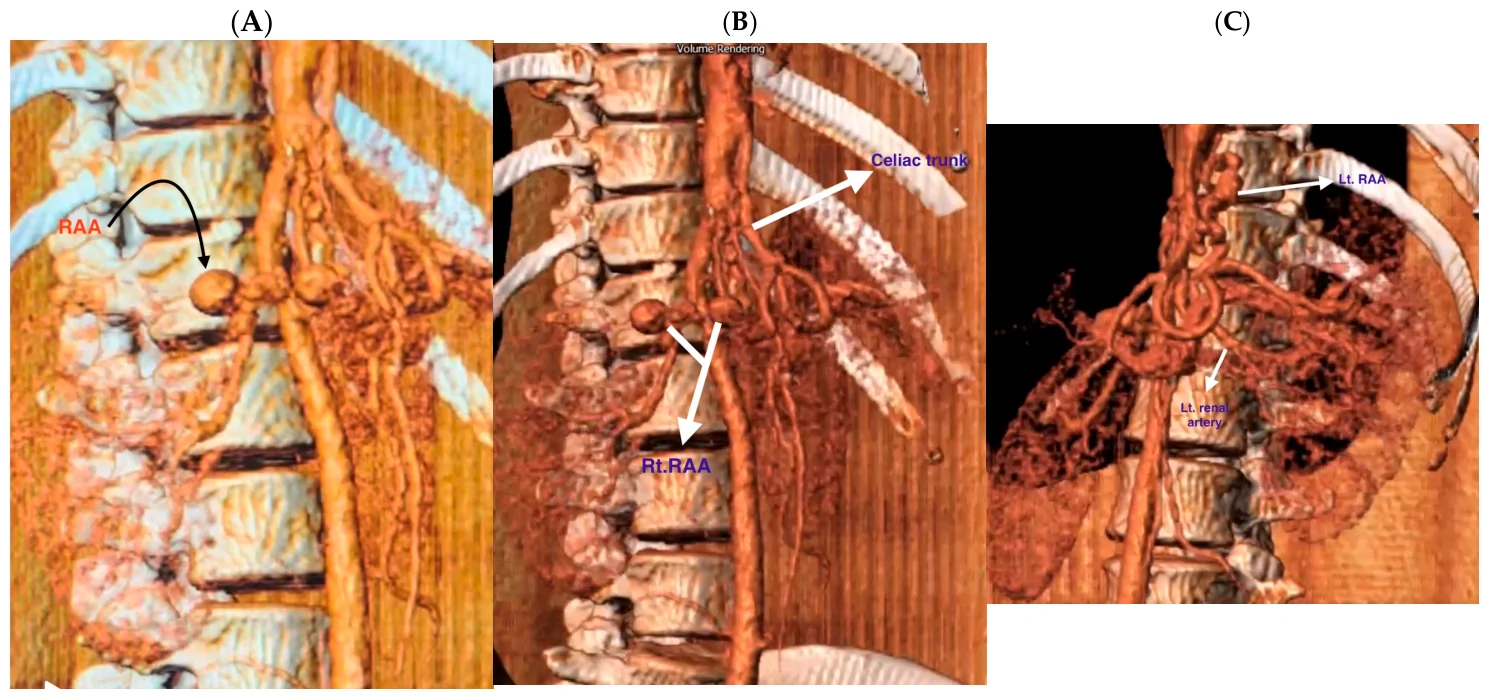
Three-dimensional computed tomography angiography volume-rendered images demonstrating the complex renal and visceral arterial anatomy. (**A**) Right renal artery aneurysm (RAA). (**B**) High-origin celiac trunk and right renal artery aneurysm. (**C**) Left renal artery and left renal artery aneurysm.

**Figure 2:**
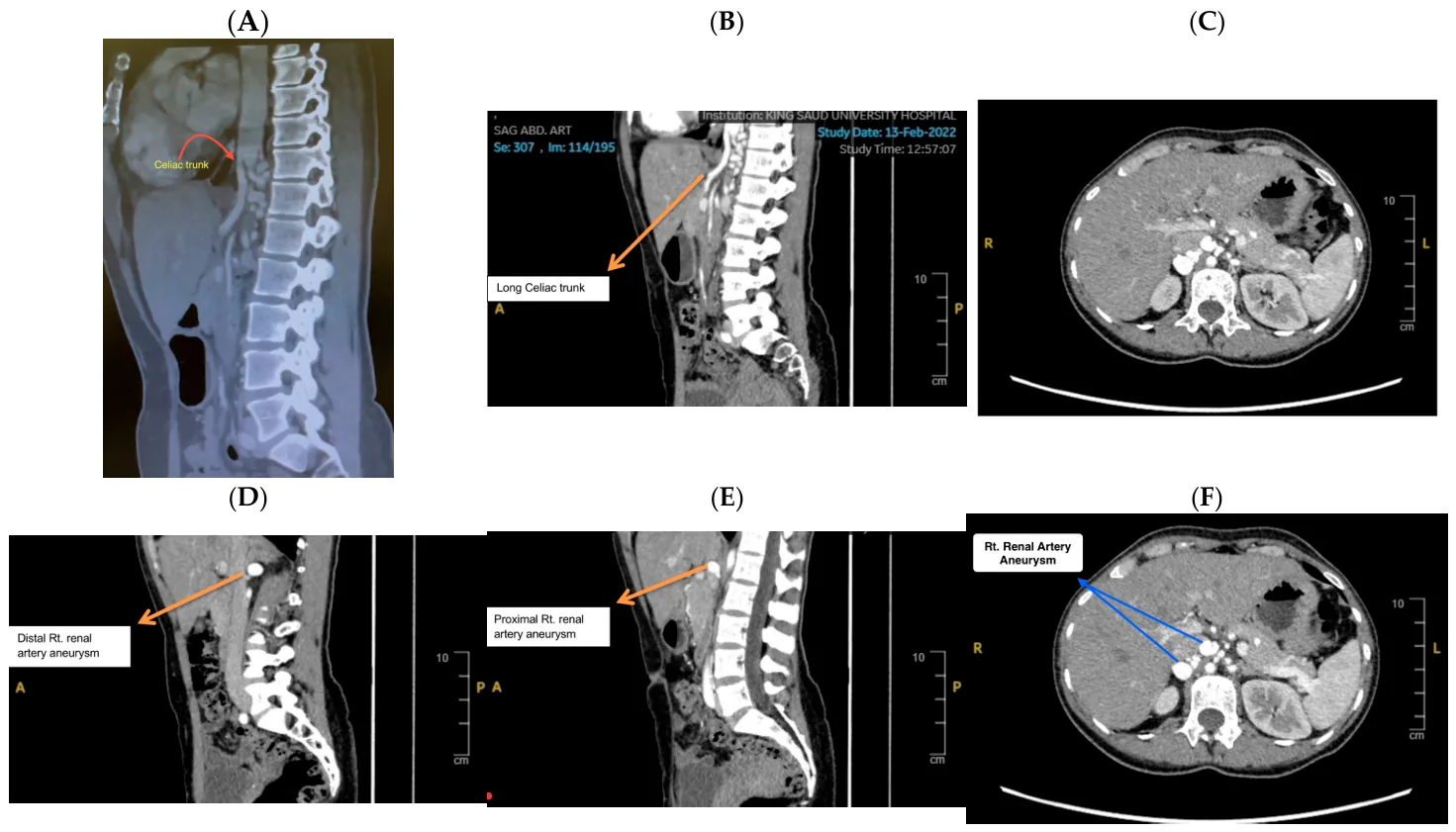
Computed tomography angiography images demonstrating the high-origin celiac trunk and right renal artery aneurysms. (**A**) Sagittal image demonstrating the celiac trunk. (**B**) Sagittal image demonstrating the long celiac trunk. (**C**) Axial image showing right renal artery aneurysmal dilatations measuring approximately 1.7 cm and 1.2 cm. (**D**) Sagittal image demonstrating a distal right renal artery aneurysm. (**E**) Sagittal image demonstrating a proximal right renal artery aneurysm. (**F**) Axial image demonstrating right renal artery aneurysms.

**Figure 3:**
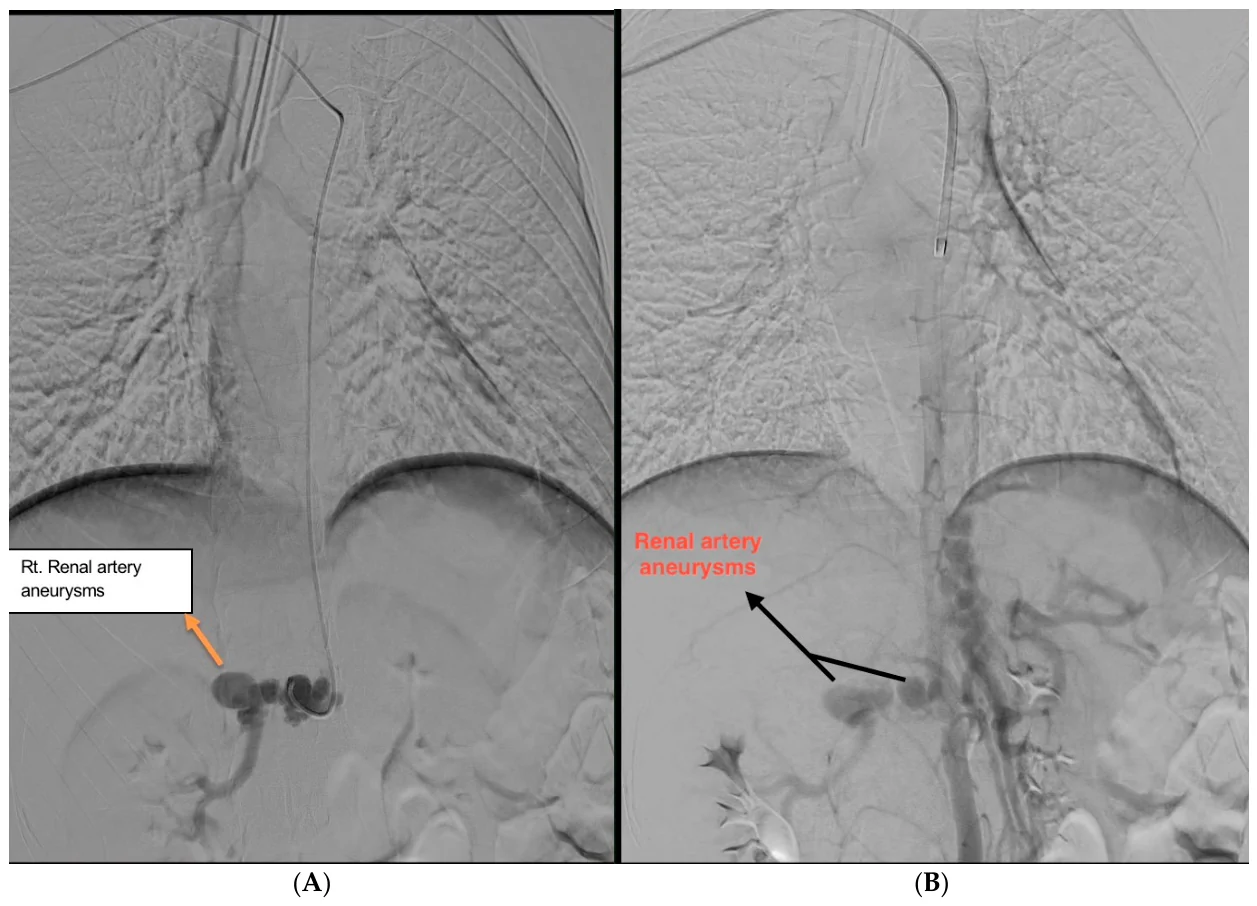
Angiographic images demonstrating multiple right renal artery aneurysms. (**A**,**B**) Representative angiographic views of the aneurysmal right renal artery.

## Data Availability

Data supporting this case report are presented within the article.

## Abbreviations

The following abbreviations are used in this manuscript:

PT	Prothrombin Time
PTT	Partial Thromboplastin Time
INR	International Normalized Ratio
RAA	Renal Artery Aneurysm
ANCA	Antineutrophil Cytoplasmic Antibodies
ANA	Antinuclear Antibody
